# Editorial: Prevalent diseases in vulnerable populations: current situation and influencing factors

**DOI:** 10.3389/fpubh.2024.1444852

**Published:** 2024-06-27

**Authors:** Yuqing Luo, Xiaolu Luo, Mulong Du, Zhuo Chen, Lu Long

**Affiliations:** ^1^Department of Epidemiology and Health Statistics, West China School of Public Health and West China Fourth Hospital, Sichuan University, Chengdu, Sichuan, China; ^2^South Railway Station Community Health Service, Chengdu, Sichuan, China; ^3^The Key Laboratory of Modern Toxicology of Ministry of Education, Department of Environmental Genomics and Genetic Toxicology, Center for Global Health, Jiangsu Key Laboratory of Cancer Biomarkers, Prevention and Treatment, Collaborative Innovation Center for Cancer Personalized Medicine, School of Public Health, Nanjing Medical University, Nanjing, China; ^4^Department of Health Policy and Management, College of Public Health, University of Georgia, Athens, GA, United States

**Keywords:** vulnerable populations, prevalent diseases, editorial, public health, demographic changes and health risks

## The concept of vulnerable populations

Vulnerable populations are more susceptible to adverse health outcomes than others because of socioeconomic status. Unlike at-risk populations, vulnerable groups face a higher overall distribution of specific risk factors (1), highlighting the importance of targeted public health interventions to mitigate the risks faced by these groups.

## Demographic changes and health risks

Since the early 21st century, many countries have experienced significant demographic shifts, including rapid aging, declining fertility rates, and increased life expectancy. These demographic changes present both opportunities and challenges for public health systems. However, social security systems have often failed to keep pace with these changes, leaving older adults particularly vulnerable to health risks. The aging population is more susceptible to chronic diseases, such as cardiovascular disease, diabetes, osteoporosis, and even cancer, which require long-term management and substantial healthcare resources. Ensuring adequate healthcare and social support for this growing demographic is crucial for maintaining their quality of life and reducing the burden on healthcare systems.

Children, another critical vulnerable group, face rising threats from infectious and chronic diseases. The COVID-19 pandemic has further exacerbated these risks, with many children experiencing psychological issues that require urgent attention from public health researchers (2). The pandemic has disrupted education, social interactions, and access to healthcare services, exacerbating the health challenges among children. Addressing these challenges necessitates a comprehensive approach that includes mental health support, access to healthcare, and educational interventions to mitigate the long-term impact on this vulnerable population.

## Evolving factors impacting health

The rapid development of society and changing lifestyles have continuously reshaped the factors affecting vulnerable populations. Changes in diet, sleep habits, and physical activity can significantly impact health, but the specific effects—positive or negative—remain an area for further exploration. For instance, the increasing prevalence of sedentary lifestyles and unhealthy diets has contributed to the rise in obesity and related chronic conditions, such as type 2 diabetes and cardiovascular diseases, among vulnerable populations. Promoting healthy behaviors, such as regular physical activity and balanced nutrition, can improve overall health outcomes and reduce the risk of chronic diseases.

Understanding these evolving factors is crucial for developing effective interventions tailored to the needs of vulnerable groups. Public health strategies must adapt to these changes and address the root causes of health disparities, such as socioeconomic inequalities, limited access to healthcare, and lack of health education. By addressing these underlying factors, we can create a more equitable healthcare system that supports the wellbeing of all individuals, particularly those in vulnerable populations.

## Case studies and research findings

Several studies have highlighted the importance of targeted interventions and interdisciplinary collaboration in improving health outcomes for vulnerable populations. For example, Godana et al. focused on adolescents in the Guji region, a particularly vulnerable population due to limited access to sexual and reproductive health education. Adolescents in this region face numerous challenges, including high rates of early marriage, teenage pregnancy, and sexually transmitted infections. Through a school-based life skills intervention, they successfully enhanced adolescents' knowledge and skills related to sexual and reproductive health, demonstrating the critical role of education in public health.

Yan et al. investigated the impact of intergenerational care on the mental health of middle-aged and older adult individuals, a group vulnerable to both physical and psychological stress. Using data from the China Health and Retirement Longitudinal Study (CHARLS), they discovered that intergenerational care significantly enhances the mental health of this demographic, highlighting the importance of family dynamics in health outcomes. Intergenerational care, which involves providing support and care across different age groups within families, can improve mental health by fostering strong family bonds, reducing feelings of isolation, and providing emotional support. However, it is important to recognize that intergenerational care can also place significant burdens on caregivers, who may experience stress and burnout as a result of their caregiving responsibilities. Therefore, policies and programs that support caregivers, such as respite care, counseling services, and financial assistance, are essential for ensuring the wellbeing of both caregivers and care recipients.

Osteoporosis is a major health concern among older adults, who are vulnerable to this condition due to aging and associated risk factors. Wang et al. emphasized the role of sex, education, knowledge, and self-efficacy in promoting osteoporosis preventive behaviors. Their research suggests that tailored intervention strategies are essential for addressing the specific needs of older adult populations vulnerable to osteoporosis. For example, educational programs that increase awareness of osteoporosis risk factors and prevention strategies, such as calcium and vitamin D supplementation, weight-bearing exercises, and fall prevention measures, can help reduce the incidence of osteoporosis-related fractures and improve the quality of life for older adult individuals.

Sexually transmitted infections (STIs) pose a significant risk to women of reproductive age, a particularly vulnerable group due to biological and social factors. Research from Perez-Fernandez et al. provided valuable insights into the correlation between early sexual activity and STIs, highlighting the need for targeted interventions to address the unique vulnerabilities of this group. Comprehensive sex education, access to contraception, and regular STI screenings are essential components of public health strategies aimed at reducing the incidence of STIs and protecting the reproductive health of women.

## Global health governance and cooperation

Strengthening international cooperation and communication is vital for tackling public health challenges and achieving global health security, especially for vulnerable populations. Continuous learning and innovation are essential to navigate the complex and evolving public health landscape. A comprehensive review by the Sun et al. School of Global Health at Shanghai Jiao Tong University and the Center for Tropical Disease Research highlighted the success of the China-Tanzania malaria control program. This collaboration demonstrates how multi-stakeholder engagement and technical assistance can enhance local health capabilities, particularly benefiting vulnerable populations in malaria-endemic regions.

The China-Tanzania malaria control program provides a model for how international collaboration can address public health challenges effectively. By sharing expertise, resources, and best practices, countries can strengthen their health systems and improve health outcomes for vulnerable populations. Technical assistance, such as training healthcare workers, providing medical equipment, and supporting surveillance systems, plays a crucial role in building local capacity to prevent and control diseases.

## Conclusion

The articles within this research project provide a thorough investigation into the current status and determinants of prevalent diseases among vulnerable populations. By fostering international collaboration and interdisciplinary research, we aim to develop more rigorous and effective strategies for disease prevention and control. Ensuring the health and wellbeing of vulnerable groups is essential for building a resilient and equitable global health landscape. Through these collective efforts, we can better address the complex health challenges of our time and improve the quality of life for all.

## Author contributions

YL: Writing – original draft. XL: Writing – review & editing. MD: Writing – review & editing. ZC: Writing – review & editing. LL: Writing – review & editing.

## References

[1] Frohlich KL Potvin L. Transcending the known in public health practice: the inequality paradox: the population approach and vulnerable populations. Am J Public Health. (2018) 98:216–21. 10.2105/AJPH.2007.114777 18172133 PMC2376882

[2] Yoshikawa H Whipps MD Rojas NM. Commentary: New directions in developmentally informed intervention research for vulnerable populations. Child Dev. (2017) 88:459–65. 10.1111/cdev.12736 28160274

